# Comparative study between Dexmedetomidine and Ondansteron for prevention of post spinal shivering. A randomized controlled trial

**DOI:** 10.1186/s12871-018-0640-3

**Published:** 2018-11-30

**Authors:** Joseph Makram Botros, Atef Mohamed Sayed Mahmoud, Safaa Gaber Ragab, Mohammed Awad Alsaeid Ahmed, Hany Maher Salib Roushdy, Hany Mahmoud Yassin, Maged Labib Bolus, Abeer Shaban Goda

**Affiliations:** 1 Department of Anesthesia, Fayoum University, Fayoum, Egypt; 2 Department of Anesthesia, Ain Shams University, Cairo, Egypt

**Keywords:** Dexmedetomidine, Intraoperative hypothermia, Ondansetron, Shivering, Spinal anesthesia

## Abstract

**Background:**

Regional anesthesia could affect the homeostatic system functions resulting frequently in perioperative hypothermia and consequently shivering. The objective of this trial was to evaluate the efficacy of dexmedetomidine and ondansetron to reduce the incidence and severity of shivering after intrathecal blocks.

**Methods:**

This randomized placebo-controlled trial included 120 patients allocated equally in three groups. All patients were anesthetized by standard intrathecal blocks for surgical procedure at lower half of the body and received one of the study drugs intravenously (IV) according to the group assignments. Group S patients (placebo) were administered saline, Group O (ondansetron) were given 8 mg ondansetron, and Group D (dexmedetomidine) were given 1 μg/kg of dexmedetomidine. Shivering incidence and scores, sedation scores, core body temperature, hemodynamic variables, and incidence of complications (nausea, vomiting, hypotension, bradycardia, over-sedation, and desaturation) were recorded.

**Results:**

The incidence and 95% confidence interval (95% CI) of shivering in group S 57.5% (42.18–72.82%) was significantly higher than that of both group O 17.5% (5.73–29.27%), *P* < 0.001 and group D 27.5% (13.66–41.34%), *P* = 0.012. However, the difference in the incidence of shivering between group O and group D was comparable, *P* = 0.425. The sedation scores were significantly higher in group D than those of both group S and group O, *P* < 0.001. Sedation scores between group S and group O were comparable, *P* = 0.19. Incidences of adverse effects were comparable between the three groups.

**Conclusion:**

Prophylactic administrations of dexmedetomidine or ondansetron efficiently decrease the incidence and severity of shivering after spinal anesthesia as compared to placebo without significant difference between their efficacies when compared to each other.

**Trial registration:**

Pan African Clinical Trial Registry (PACTR) under trial number (PACTR201710002706318). 18-10-2017. ‘retrospectively registered’.

## Background

Unintentional hypothermia is defined as central blood temperature below 36 °C. Direct inhibition of thermoregulation by anesthetics, decreased metabolism, patient contact to the cold atmosphere of operating rooms (OR), and body cavity exposure are the main causes of perioperative hypothermia [1]. Change in temperature signals are processed at the level of the hypothalamus anteriorly, and synchronize the peripheral data with the set-point (threshold value). Temperatures greater than that set-point will initiate reactions to lower the body temperature, while temperatures lesser than that set-point will propagate responses to increase the body temperature [2, 3]. Both regional and general anesthesia are recognized to disturb the competence of that homeostatic system and may end in various grades of hypothermia. Intrathecal block drops this set-point by 0.5 °C leading to vasoconstriction and shivering above the level of the block [4]. This fall in the threshold is directly proportional to the sum of blocked spinal segments, high-level spinal blockade, and older ages [5]. Shivering results in augmentation of metabolic activity and increase in oxygen consumption leading to arterial hypoxia that may result in increased risk of myocardial ischemia, increased intracranial pressure, and intraocular pressure. The added effects are increasing in cardiac output, peripheral vascular resistance, carbon dioxide production and lactic acidosis [6, 7]. Furthermore, shivering impedes electrocardiogram (ECG) and peripheral oxygen saturation (pulse oximetry) monitoring [8]. Tympanic temperature monitoring by using tympanic probe is one of the best reliable methods and the gold standard of core temperature monitoring [9]. Perioperative hypothermia and shivering are usually prevented by physical methods like surface warming or pharmacologically by administration of pethidine, clonidine, or tramadol [1–4]. Nonetheless, the efficacy and safety findings remain unclear or even inconsistent [10].

Ondansetron is a 5HT 3 (5-hydroxytryptamine 3) receptor antagonist, primarily used for management emesis. Recently, it has also been tried effectively for prevention of shivering with favorable safety profile [11]. Dexmedetomidine, a powerful *α* 2-adrenergic receptor agonist, has been used as a sedative agent and is documented to increase the shivering threshold [12]. There are few studies evaluating the use of prophylactic dexmedetomidine and ondansetron separately for prevention of shivering during spinal anesthesia, while there are no studies that directly compare the two drugs. The aim of this trial was to evaluate and compare directly the relative efficacy and safety of ondansetron and dexmedetomidine for decreasing the incidence and severity of shivering after spinal anesthesia.

## Methods

The trial design was a prospective, double-blinded, randomized, parallel three arm groups, and placebo-controlled study. The local ethical committee approval was obtained from Fayoum University Hospitals (R 64) and the study protocol was registered in Pan African Clinical Trial Registry (PACTR) under trial number (PACTR201710002706318). Each participant was informed about the study protocol in details and complete written informed consents were signed before enrollment in the study. One hundred twenty patients of both genders aged 18–60-years ASA physical status I&II, undergoing elective surgical procedures in the lower half of the body (orthopedic, general or gynecological surgeries) were included in this trial. Surgical procedures included hernioplasty, appendicectomy, vaginal hysterectomy, abdominal hysterectomy, or open reduction and internal fixation of lower limbs. Patients excluded were those with thyroid disorders, patients with a history of convulsions, multiple allergies, severe cardiopulmonary diseases, pregnancy, uncooperative patients, patients requiring blood transfusion, and patients with severe hepatic or renal diseases. The selected patients were randomly allocated using computer generated method and opaque sealed envelopes into 3 groups containing 40 patients each according to the study drug; Placebo or Saline group (Group S), Ondansetron group (8 mg) (Group O), and dexmedetomidine group (1 μg/kg) (Group D). Preoperatively, demographic characteristics as age, sex, height, and weight were recorded.

After admission to the OR, routine standard monitoring was used in all patients in the form of non-invasive blood pressure (NIBP), pulse oximetry and ECG. The temperature of the operating room was maintained at 24 °C–26 °C by adjusting the temperature setting of air conditioners. Before intrathecal block, each patient was preloaded with 15 ml/kg of Ringer Lactate solution. The block was introduced at either L3/4 or L4/5 interspace with 3 ml of 0.5% hyperbaric bupivacaine (15 mg) by attending anesthesiologists who were not participating in this trial. After completion of intrathecal blocks, the patient lied supine and oxygen was administered via a nasal cannula (2 L/min) till the end of the procedure. Tympanic membrane temperature was monitored with Braun® thermoscan thermometer every 5 min for 45 min after the intrathecal block. The intravenous fluids were kept at room temperature (24 °C- 26 °C) and all the patients were covered with a standard single blanket. Just after the intrathecal injection, one of the study drugs was given slowly by IV route over five minutes. The study drugs were prepared, diluted to a volume of 5 ml and presented as coded syringes by an anesthetist who was not involved in the management of the patients or data acquisition.

During and shortly after completion of the surgical procedures, the data of non-invasive blood pressure, heart rate, oxygen saturation, core body temperature, duration of surgical procedures, type of surgical procedures and the level of intrathecal blocks were recorded.

The primary outcome was the incidence of shivering in the early 45 min after intrathecal blocks as defined by a shivering score ≥ 3 at any time of the predefined assessment points (highest score). Shivering score, sedation score, incidence of hypotension, incidence of bradycardia, incidence of hypoxemia, and incidence of nausea, and vomiting were secondary outcomes. The shivering score were assessed at 5 min interval for 45 min after intrathecal block and graded using a scale like that validated by Tsai and Chu [15] (Grade 0: no shivering, Grade 1: piloerection or peripheral vasoconstriction but no visible shivering, Grade 2: muscular activity in only one muscle group, Grade 3: muscular activity in more than one muscle group but not generalized and Grade 4: shivering involving the whole body). Continuous shivering ≥ grade 3 for 15 min was considered significant side effect of intrathecal block despite prophylactic IV administration of study drugs and a rescue dose of 0.5 mg / kg of pethidine was administered to control this unpleasant prolonged shivering. Sedation scores (highest score) were assessed on 5-point scale (1: fully awake and oriented patient, 2: drowsy, 3: eyes closed, arousable on command, 4: eyes closed, arousable to physical stimuli, 5: eyes closed and patient unarousable to physical stimuli). Over-sedation was defined as sedation score ≥ 4 with hypoxemia (oxygen saturation < 92%), necessitate conscious level monitoring, or require postoperative intensive care admission and its incidence was recorded. Hypotension (systolic blood pressure < 90 mmHg) was controlled by IV ephedrine administration 5 mg increments and by IV fluid boluses to keep systolic blood pressure ≥ 90 mmHg upon the discretion of the attending anesthesiologists. Bradycardia (heart rate < 60 beats/ minute) was treated by IV atropine sulphate 10 μg/kg upon the judgment and preferences of the attending anesthesiologist. Nausea and vomiting incidences were recorded and managed according to the attending anesthesiologist discretion. The investigators who were responsible for data collection and analysis were blinded to the groups’ allocation and all patients and care givers were unaware of the administered IV study drugs nature. The current study adheres to CONSORT guidelines.

### Statistical analysis of data

A pilot study was performed prior to patient recruitment to estimate an appropriate sample size. The pilot study encompassed 45 subjects (15 in each arm). The incidence of shivering (the primary outcome) in placebo **S** group was 40% (6 subjects), in group **O** was 13% (2 subjects), and in the group, **D** was 20% (3 subjects). A sample size of 33 participants was determined per group by using a Z test family, assuming two tail *α* = 0.05, 80% power (*β* = 0.2), and an allocation ratio = 1. Recruitment of 40 participants per group was done to account for possible protocol violation or data loss. Sample size calculation was estimated by using G*Power software version 3.1.9.2 (Institute of Experimental Psychology, Heinrich Heine University, Dusseldorf, Germany).

Continuous parametric data were presented as mean ± standard deviation (SD), ordinal non-parametric data were presented as median (interquartile range) (IQR), and categorical data were presented as number of patients and proportions. Shapiro Wilk test was used to test the normality of data distributions, *P* < 0.05. Continuous variables including hemodynamic data and temperature values were analyzed using repeated measures analysis of variance followed by Bonferroni’s post-hoc testing to determine intragroup, within groups, and groups by time differences. Shivering and sedation scores between three groups were compared using the Kruskal–Wallis test and post hoc test was done by implementation of the Dunn’s test. Nominal data were analyzed and compared using the Chi-square test or Fisher exact test when appropriate.

Adjusted *P* values for multiple post-hoc comparisons between every 2 group were calculated by using the Bonferroni correction method to account for the problem of multiple testing (type I error inflation). The *P*-value of 0.05 was divided by the number of comparisons i.e. 3 (0.05/3). Thus, test result with *P* values < 0.017 were considered statistically significant difference for multiple post-hoc comparisons between every 2 groups otherwise, *P*-values of < 0.05 were considered statistically significant. Statistical analysis was performed using the SPSS version 17 (Inc., Chicago, IL, USA).

## Results

One hundred twenty recruited patients completed the study protocol successfully (40 patients in each group) without exclusion of any participants and data analysis were done per protocol. The difference between the three groups in demographic characteristics, surgical procedure duration, type of surgical procedures and intrathecal block level were statistically comparable. (Table 1).

**Table 1:** Demographic characteristics and Operative data

Parameters	Group S’ (*n* = 40)	Group O (n = 40)	Group D (n = 40)	*p*-value
Age (years)	33.88 ± 7.8	33.9 ± 9.85	37.2 ± 9.84	
Sex (M/F)	21/19	24/16	21/19	
Weight (Kg)	79.25 ± 9.75	79.9 ± 9.9	81.8 ± 11.2	
Height (meter)	1.64 ± 0.16	1.63 ± 0.24	1.64 ± 0.18	0.67
Duration of surgeries (min)	82.65 ± 15.2	85.24 ± 10.17	84.02 ± 12.45	0.32
Intrathecal block level (thoracic segment)	T8 (8–10) 6–10	T10 (8–10) 8–10	T8 (8–10) 8–10	0.22
Types of surgical Procedures n (%)				0.932
• Orthopedics	18(45%)	21(52.5%)	17(42.5%)	
• Hernioplasties	11(27.5%)	11(27.5%)	14(35%)	
• Vaginal Hysterectomies	5(12.5%)	3(7.5%)	6(15%)	
• Appendectomies	4(10%)	3(7.5%)	2(5%)	
• Abdominal Hysterectomies	2(5%)	2(5%)	1(2.5%)	

Data are presented as mean ± SD or median (IQR), or number (percentage)

٭ *P* < 0.001 is considered statistically significant

**Fig. 1:**
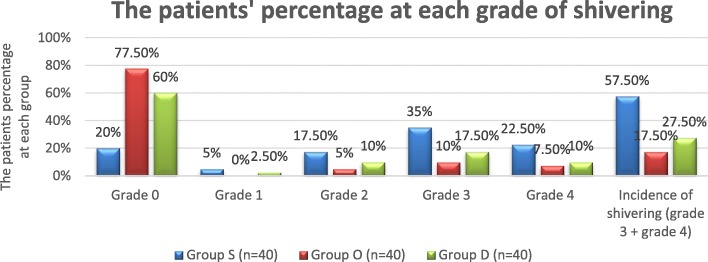
The percentage of patients at each grade of shivering among the three groups. Group S: Saline group, Group O: Ondansetron group, Group D: Dexmedetomidine group

The incidence and 95% confidence interval (95% CI) of shivering in group S 57.5%(42.18–72.82%) was significantly higher than that of both group O 17.5%(5.73–29.27%), *P* < 0.001 and group D 27.5% (13.66–41.34%), *P* = 0.012. However, the difference in the incidence of shivering between group O and group D was statistically and clinically insignificant, *P* = 0.425. (Fig. 1).

There were no statistically significant differences between the three groups in scores of shivering at 5, 40, and 45 min after intrathecal block, *P* = 1.0, 0.167, and 1.0 respectively. Shivering scores in group S were significantly higher than that of both groups O and D at time-points 10 to 35 min after intrathecal blocks, *P* < 0.01. The differences of shivering scores between group O and group D were comparable during the same time-points, *P* > 0.05. There were no reported cases of prolonged uncontrolled shivering in any of the three groups and pethidine doses were not used in any of the recruited cases as a rescue agent.

**Fig. 2:**
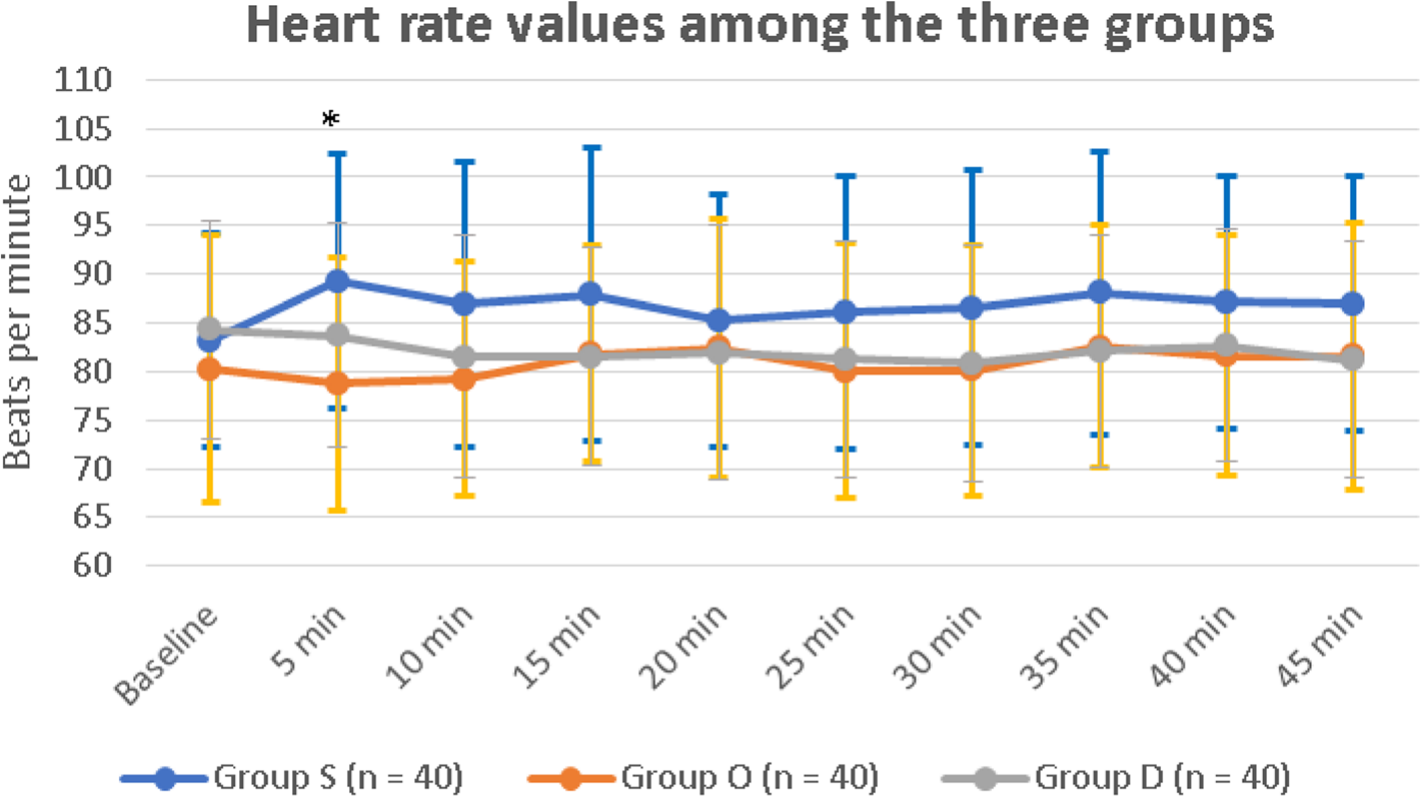
The heart rate values among the three groups. Group S: Saline group, Group O: Ondansetron group, Group D: Dexmedetomidine group. *P < 0.001

The sedation scores were significantly higher in group D than those of both group S and group O, *P* < 0.001. Sedation scores between group S and group O were comparable, *P* = 0.19. (Table 2) There were no reported cases of over-sedation (score ≥ 4) could be determined at any of the predefined assessment time-points that led to hypoxemia, necessitated conscious level monitoring, or required postoperative intensive care admission. The heart rates values were significantly different between the three groups only after 5 min of intrathecal blocks, *P* < 0.001. These values were comparable between group O and group D, *P* = 0.071 and also comparable between group S and group D, *P* = 0.0495. While the difference was statistically significant between group S and group O, *P* = 0.0005. Otherwise, there were no statistically significant differences between the three groups at any of the other predefined time-points of assessments. (Fig. 2) The incidence of bradycardia was comparable between the three groups. (Table 2).

**Table 2:** Sedation Scores, Incidences of complications, and Incidence of ephedrine and atropine Use

Parameters	Group S (n = 40)	Group O (n = 40)	Group D (n = 40)	*p*-value
Sedation Scores	0(0–0) 0–4	0(0–0) 0–2	1(1–1) 1–2	< 0.001٭
Incidence of hypotension n (%)	15(37.5%)	14(35%)	15(37.5%)	0.97
Incidence of bradycardia n (%)	10(25%)	9(22.5%)	9(22.5%)	0.955
Incidence of nausea and vomiting n (%)	10(25%)	3(7.5%)	7(17.5%)	0.094
Number of patients treated with ephedrine n (%)	10(25%)	9(22.5%)	10(25%)	0.675
Number of patients treated with ephedrine and atropine n (%)	1(2.5%)	0(0%)	0(0%)	0.87

Data are presented as median (IQR)(range) or as number of patients (percent)

٭ *P* < 0.001 is considered statistically significant

**Fig. 3:**
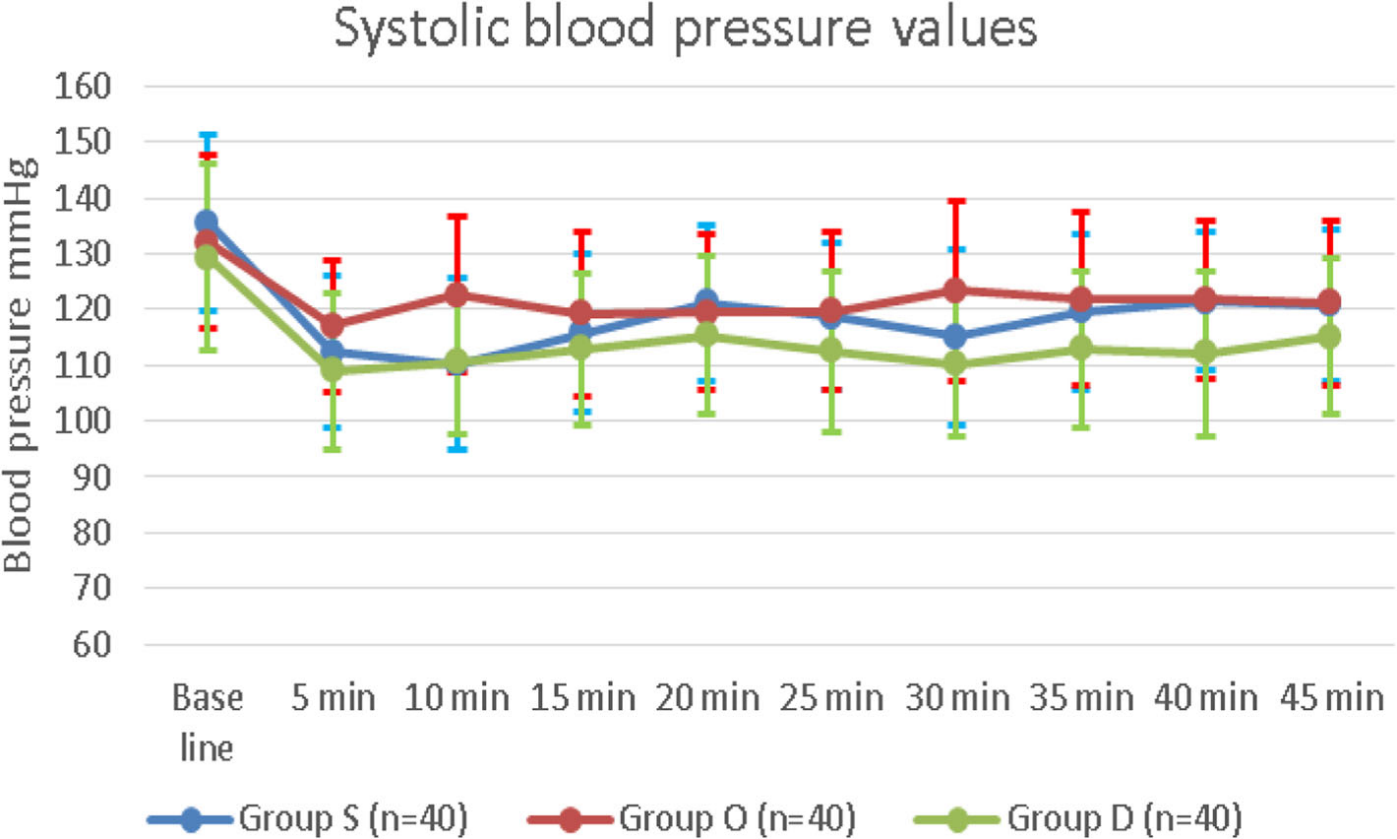
Systolic blood pressure values among the three groups Group S: Saline group, Group O: Ondansetron group, Group D: Dexmedetomidine group

**Fig. 4:**
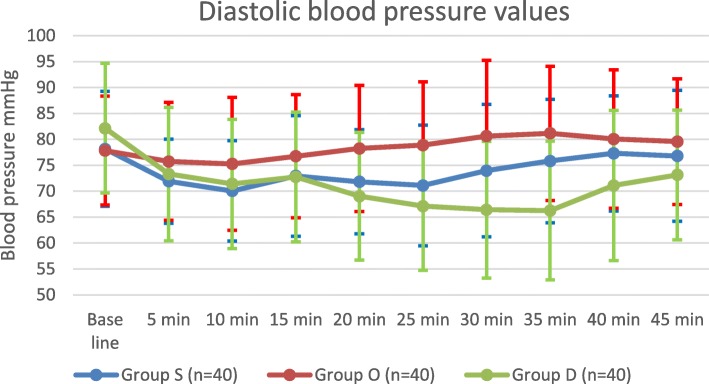
Diastolic blood pressure values among the three groups. Group S: Saline group, Group O: Ondansetron group, Group D: Dexmedetomidine group

**Fig. 5:**
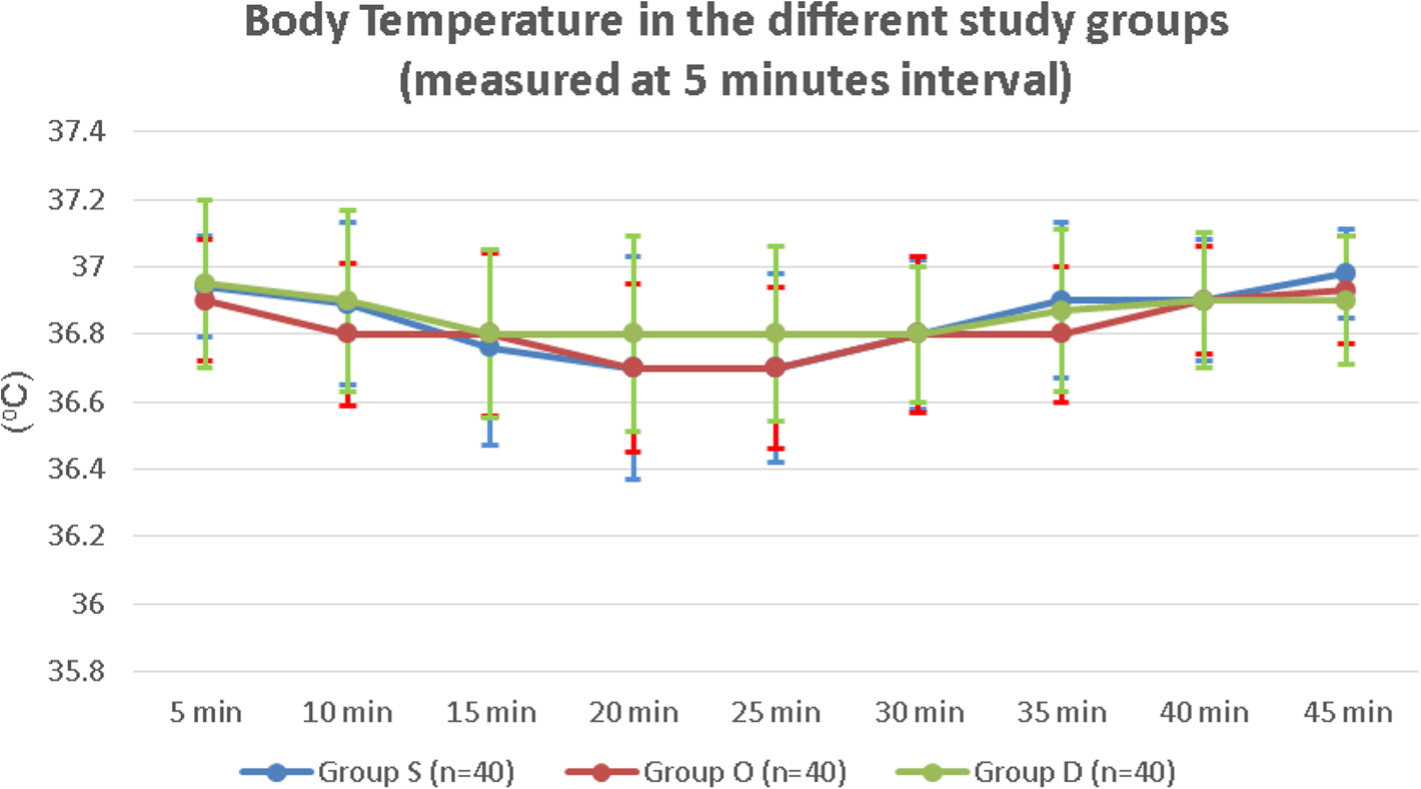
Body temperature measurements at the predefined time points between the three groups. Group S: Saline group, Group O: Ondansetron group, Group D: Dexmedetomidine group

There were no statistically significant differences between the values of systolic blood pressure (SBP) (Fig. 3), diastolic blood pressure (DBP) (Fig. 4), incidence of hypotension, and incidence of nausea and vomiting of the three groups. (Table 2) There were no reported cases of hypoxemia in any of the recruited patients. The body temperature values were comparable among the three groups during all the time-points. There were no detected intragroup variability, or group by time interaction. (Fig. 5).

## Discussion

The finding of the current trial verifies the efficacy and safety of the prophylactic distinct use of drug, ondansetron or dexmedetomidine, in reducing the incidence and severity of the shivering that accidently arisen after intrathecal blocks when compared to the placebo. The accompanying side effects related to the use of these drugs were transitory, self-limited, and easily tolerable by most of the recruited subjects.

literatures defined shivering as an unintentional muscular action that augments heat generation aiming to restore homeostasis and suggested that the vasoconstriction threshold is 1 °C higher than the shivering threshold [13–15]. As hypothermia continues, the shivering threshold is attained and motor neurons are employed for heat production with increasing magnitude [16]. The end effect of this autoregulatory process is to increase heat genesis up to 600% and triples oxygen depletion [7]. Nevertheless, these responses are temporary [17]. The total heat production generated from skeletal muscle contraction is mostly modest, as much of these energy will not be reserved in the body core but probably it will be lost to the surroundings. The increase in core body temperature will be only about 1 °C. Shivering could be prevented and controlled by adjusting OR temperature, warming IV fluids, using warming blankets, or by administrating IV drugs [1–4].

The findings of the current trial go in agreement with the results of Shakya and colleagues who showed that shivering was 10% only in ondansetron group compared to 42.5% in placebo group [18]. They concluded that the prophylactic administration of ondansetron showed a substantial reduction of the incidence and scores of shivering when compared to placebo [18]. Nallam and colleagues compared the prophylactic effect on shivering of IV ondansetron versus placebo on 80 parturients undergone lower segment cesarean section under spinal anesthesia [19]. They found statistically significant difference between the study groups and concluded that ondansetron could be used safely in this particular group of patients to prevent shivering [19]. Kelsaka et al. and Badawy et al. highlighted the importance of ondansetron as a prophylactic agent in shivering prevention after intrathecal blocks [20, 21]. In contrast to the findings of the current study, Browning and colleagues found no significant difference in the occurrence of shivering between those who received ondansetron (8 mg) and those who received saline as a placebo [22]. This could be attributed to the different scoring system they have used, and intrathecal fentanyl administration in their study groups. The supposed mechanism of action of ondansetron as an anti-shivering agent is not clear and it is anticipated to act centrally by inhibition of serotonin reuptake at the level of the pre-optic anterior hypothalamic region [20].

Bajwa and colleagues emphasized the anti-shivering effect of dexmedetomidine as the incidence of shivering in dexmedetomidine group was 5% compared to 42.5% in the control group of their trial. Their findings go in line with the findings of the current trial [23]. Megalla and colleagues found that dexmedetomidine produced a rapid and effective control of shivering in 100% of patients compared to 92% of patients in nalbuphine group and 32% in the placebo group. They observed statistically significant difference only when dexmedetomidine group and nalbuphine group were compared to placebo group [24]. Their study differed from the current study in using lower dose of dexmedetomidine (0.5 μg/kg) and the participants in their trial were only candidates of vaginal hysterectomies under spinal anesthesia [24]. Dexmedetomidine exerts its anti-shivering effect probably through a central *α-*2 agonistic effects which may carry the risk of certain side effects as over-sedation, dangerous bradycardia, and hypotension [25].

The findings of the current trial showed that the patients in dexmedetomidine group had a higher level of sedation scores compared to other groups, *P* < 0.001. (Table 2) It is noteworthy that there were no reported cases of over-sedation at any of the study groups and these results are in accordance also with the findings of Bajwa and colleagues [23].

The findings of the current trial showed that there was a statistically significant difference between group S and group O only after 5 min of intrathecal blocks in heart rate. (Fig. 2) This could be attributed to the relative heart rates increase in placebo group as a reflex to spinal anesthesia induced hypotension despite comparable values of blood pressure values between the three groups at this time-point of assessment. It is noteworthy that ondansetron may pose protective potentials against spinal anesthesia induced hypotension [26, 27]. In contrast; Zhou and colleagues concluded that ondansetron had no actual capabilities to reduce the incidence of hypotension and shivering during cesarean section after spinal anesthesia, but could efficiently decrease the incidences of nausea, vomiting, and bradycardia [28].

The incidences of adverse events in the current trial (hypotension, bradycardia, nausea, and vomiting) were comparable between the three groups denoting the high safety profile of both drugs when compared against the placebo [19–29].

The use of pethidine, the gold standard anti-shivering drug, and other opioid drugs in management of perioperative shivering could be associated with opioid related side effects as over-sedation, respiratory depression, nausea and vomiting, itching, constipation, and postoperative opioid induced hyperalgesia [30]. Both study drugs devoid these unfavorable responses, but may in addition limit the occurrence and frequency of most of such adverse effects.

The finding of the current trial is limited by inherent elements in its design. The validity of its results could not be suitable in other types of anesthesia other than intrathecal blocks, other types of extensive surgical interventions that mandates large fluid shifts and huge third space losses, or endoscopic Urosurgical procedures that are accompanied by irrigation of large amounts of fluid that may diffuse to body compartments rapidly and affect the thermoregulatory mechanisms leading to marked hypothermia and consequently uncontrolled shivering. The generalizability of these study findings was limited to subjects who have normal thyroid, cardiac, renal and hepatic functions.

## Conclusion

In conclusion, the current trial demonstrated that prophylactic administrations of dexmedetomidine or ondansetron efficiently decrease the incidence and severity of shivering after spinal anesthesia as compared to placebo with tolerable adverse effects.

## Abbreviations

5HT 3: 5-Hydroxytryptamine 3
DBP: Diastolic blood pressure
ECG: Electrocardiogram
IQR: Interquartile range
IV: Intravenous
NIBP: Non-invasive blood pressure
SBP: Systolic blood pressure
SD: Standard deviation
